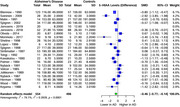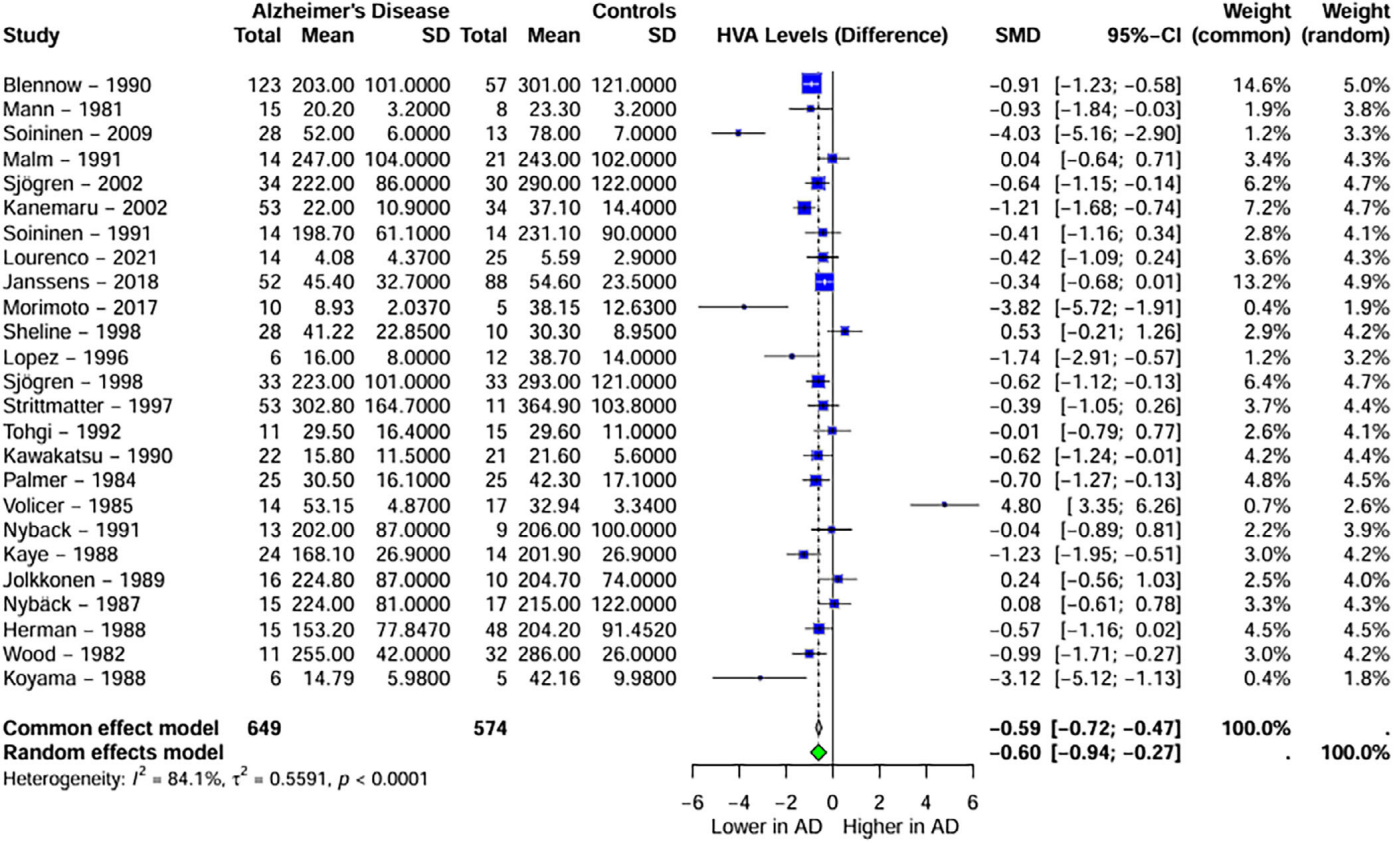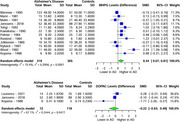# Alterations in Monoamine Metabolites in Alzheimer's Disease: A Comprehensive Meta‐Analysis of Cerebrospinal Fluid Levels

**DOI:** 10.1002/alz70856_100217

**Published:** 2025-12-24

**Authors:** Muneeb Ahmad Muneer, Harshita Agarwa, Poorvikha Gowda, Mohammad Orooj Azmi, Parshant Yadav, Victor Ghosh, Mohammad Hassan, Anmol Kaur, Vinay Suresh, Ahmed Y. Azzam, Mainak Bardhan, Alzheimer's Interest and Analytics Group ‐ NeuroStats Network

**Affiliations:** ^1^ Allama Iqbal Medical College, Lahore, Punjab, Pakistan; ^2^ Institute of Post Graduate Medical Education and Research, Kolkata, West Bengal, India; ^3^ St John's Medical College, Bangalore, Karnataka, India; ^4^ Maulana Azad Medical College, New Delhi, Delhi, India; ^5^ Andhra Medical College, Visakhapatnam, Andhra Pradesh, India; ^6^ Katihar Medical College, Katihar, Bihar, India; ^7^ Lady Hardinge Medical College, New Delhi, Delhi, India; ^8^ King George's Medical University, Lucknow, Uttar Pradesh, India; ^9^ Montefiore‐Einstein Cerebrovascular Research Lab, Albert Einstein College of Medicine, New York, NY, USA; ^10^ Miami Cancer Institute, Baptist Health South Florida, USA, Miami, FL, USA

## Abstract

**Background:**

Alzheimer's disease (AD) is a neurodegenerative disorder marked by cognitive decline and neuropsychiatric symptoms. The monoaminergic system, crucial for mood, cognition, and behavior regulation, is implicated in AD pathology. Alterations in monoamine metabolites like 3,4‐dihydroxyphenylacetic acid (DOPAC), 5‐hydroxyindoleacetic acid (5‐HIAA), 3‐methoxy‐4‐hydroxyphenylglycol (MHPG), and homovanillic acid (HVA) have been linked to cognitive decline. This meta‐analysis compares cerebrospinal fluid (CSF) levels of these metabolites in AD patients and healthy controls, offering insights into AD pathogenesis and potential therapeutic targets.

**Method:**

We searched MEDLINE, EMBASE, Cochrane, and Scopus for studies on monoamine metabolite concentrations in AD patients' CSF, following PRISMA guidelines. The meta‐analysis was conducted using R's 'meta' package with inverse variance weighting, calculating mean concentrations and standardized differences. Standardized Mean Differences (SMD) were used as the primary outcome measure. Heterogeneity was assessed with I^2^ and tau^2^ values, and tau^2^ was estimated using restricted maximum‐likelihood and Q‐profile methods.

**Result:**

In our analysis of four monoamine metabolites across AD patients and healthy controls, distinct alterations were observed. 5‐HIAA showed a significant SMD of ‐0.46 (95% CI: ‐0.77 to ‐0.14, I^2^ = 79.1%) in 22 studies (554 AD, 496 controls). HVA exhibited a significant SMD of ‐0.60 (95% CI: ‐0.94 to ‐0.27, I^2^ = 84.1%) in 25 studies (649 AD, 574 controls). MHPG presented a significant SMD of 0.44 (95% CI: 0.07 to 0.81, I^2^ = 75.4%) in 12 studies (319 AD, 305 controls). Meanwhile, DOPAC showed a non‐significant SMD of ‐0.22 (95% CI: ‐0.93 to 0.49, I^2^ = 67.1%) in 3 studies (72 AD, 118 controls).

**Conclusion:**

5‐HIAA and HVA concentrations were significantly lower in AD patients compared to healthy controls, suggesting dysfunction in the serotonergic and dopaminergic systems. Conversely, MHPG levels were elevated, pointing to potential increased norepinephrine breakdown. DOPAC levels did not show a significant difference. These results underscore the dysregulation of monoaminergic neurotransmission in AD, with the need for further studies to validate and utilize these findings for potential therapeutic target identification.